# The effect of high-fat diet on rat’s mood, feeding behavior and response to stress

**DOI:** 10.1038/tp.2015.178

**Published:** 2015-11-24

**Authors:** S Aslani, N Vieira, F Marques, P S Costa, N Sousa, J A Palha

**Affiliations:** 1 Life and Health Sciences Research Institute (ICVS), School of Health Sciences, University of Minho, Braga, Portugal; 2 Life and Health Sciences Research Institute (ICVS)/3B’s - PT Government Associate Laboratory, Guimarães, Braga, Portugal

## Abstract

An association between obesity and depression has been indicated in studies addressing common physical (metabolic) and psychological (anxiety, low self-esteem) outcomes. Of consideration in both obesity and depression are chronic mild stressors to which individuals are exposed to on a daily basis. However, the response to stress is remarkably variable depending on numerous factors, such as the physical health and the mental state at the time of exposure. Here a chronic mild stress (CMS) protocol was used to assess the effect of high-fat diet (HFD)-induced obesity on response to stress in a rat model. In addition to the development of metabolic complications, such as glucose intolerance, diet-induced obesity caused behavioral alterations. Specifically, animals fed on HFD displayed depressive- and anxious-like behaviors that were only present in the normal diet (ND) group upon exposure to CMS. Of notice, these mood impairments were not further aggravated when the HFD animals were exposed to CMS, which suggest a ceiling effect. Moreover, although there was a sudden drop of food consumption in the first 3 weeks of the CMS protocol in both ND and HFD groups, only the CMS-HFD displayed an overall noticeable decrease in total food intake during the 6 weeks of the CMS protocol. Altogether, the study suggests that HFD impacts on the response to CMS, which should be considered when addressing the consequences of obesity in behavior.

## Introduction

Obesity is one of the main health concerns in today’s society.^1^ It is mainly described as an excessive increase in body weight, with disproportionate accumulation of body fat mass, caused by excess energy intake over energy expenditure over a long period of time.^1, 2^ Besides the well-recognized set of metabolic alterations, obesity is also suggested to be associated with psychiatric disorders, such as anxiety and depression^2^ (albeit not all studies show such association).^3, 4, 5^ Depression in itself is a serious chronic mental disorder characterized by health complications including, among others, anhedonia and changes in appetite and pattern of food intake.^6, 7, 8, 9^ Mild environmental stressors, to which individuals are exposed daily, can be both triggers of depression and alter feeding behaviors.^1, 2^ Thus, these may, directly or indirectly, contribute to the metabolic changes triggered by elevated glucocorticoids, as well as to the onset of obesity.^1, 10, 11^ However, it is interesting to note that the individual’s response to stress depends on various factors, including the type of stressors, their intensity, frequency and duration.^12^ In fact, the individual specificities in the stress response, namely in terms of metabolism, may explain some of the controversies in the literature. Studies in animal models of stress showed that while intense and painful stressors result in inhibition of food intake,^12^ and exposure to chronic mild stressors leads to a reduction in food intake,^1, 2, 12, 13^ other mild stressors induce spontaneous feeding.^1^ Still, most of the studies have focused on the acute effect of stress on animals’ feeding behavior and are mostly conducted using healthy animals displaying normal body weight and fed with regular rodent diet. Therefore, information is lacking on the effects of stress in animal models under other types of diets, namely high-fat diet (HFD). Here we used a model of HFD-induced obesity to evaluate the consequences of HFD on mood and cognition and on the animals’ ability to respond to chronic mild stress (CMS).

## Materials and methods

### Animals and diets

Animals were housed under standard laboratory conditions (two rats per cage; room temperature 22 °C and humidity of 55% light change every 12 h with lights on at 0800 hours; food and water *ad libitum*). All animals were used in accordance with European Union regulations (Directive 86/609/EEC); and the experimental protocol was approved by the national competent authority, Direção-Geral de Alimentação e Vererinária (DGAV). Male offspring from eight pregnant Wistar Han rats (Charles-River Laboratories, Barcelona, Spain) were separated from their mothers at the age of 3 weeks and randomly divided in two groups with different diets: (i) normal rat laboratory chow (normal diet (ND), total 2.67 kcal g^−1^—carbohydrate 53.5%, fat 3% and protein 18.5% diet #4RF21-GLP certificate, Mucedola, Milan, Italy); (ii) obesity-inducing diet, HFD (total 4.73 kcal g^−1^—carbohydrate 35%, fat 45% and protein 20% diet #D12451, Research Diet, New Brunswick, NJ, USA). HFD is commonly used to induce obesity.^14, 15^ ND allows for comparisons both with the HFD animals, and also with data obtained from our and other laboratories on the effect of CMS (which is done in animals fed with ND).

**Figure 1:**
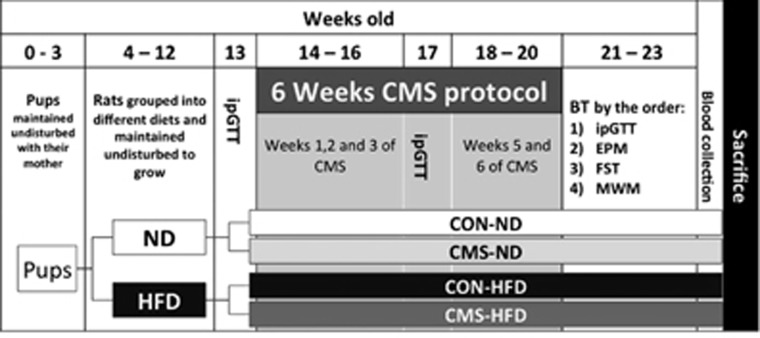
Experimental timeline. Rats were sorted into two groups with different diets, immediately after weaning until 13 weeks of age and thereafter separated into 2 additional subgroups of CON and CMS for each diet. Behavioral tests were performed, in the order shown, immediately after the CMS protocol (6 weeks). All tests were performed in the light phase of animals’ diurnal light cycle. Blood was collected in the two light phases, 5 days after the last behavioral test was performed. CMS, chronic mild stress; CON, control; EPM, elevated plus maze; FST, forced swimming test; HFD, high-fat diet; ipGTT, i.p. glucose tolerance test (at 3 time points); MWM, Morris water maze; ND, normal diet.

Rats were kept undisturbed until they reached 13 weeks of age, and thereafter each group (ND and HFD) was further randomly sub-divided into two groups, one control (CON) and one exposed to CMS. The sample size for all the experiments was of *n*=11 for each CON group, *n*=8 for each CMS-ND group and *n*=12 for each CMS-HFD group, except for the corticosterone evaluation (*n*=6 CON-ND, *n*=7 the rest of the groups; corresponding to the number of animals for which serum was available). The body weight (individual rat) and food intake (pair of rats of each cage) of all animals were measured weekly over the course of the experiment. CON groups were handled for ~10 min, 3 times per week, throughout the experiment. Handlings were performed by holding each rat on the experimenters’ arm or by allowing the rat to explore the environment outside the cage, mainly on the bench around their cage. A battery of behavioral tests (BT) was performed immediately after 6 weeks of exposure to CMS. BTs were performed in the animals’ light phase and in the following specific order, according to the sensitivity of the test, to avoid potential between-test interference effects: elevated plus maze (EPM), forced swimming test (FST) and Morris water maze (MWM).^7, 16^ Experiments were performed twice; and results are representative of one independent experiment. All BTs and corticosterone level analysis were performed blinded to the researcher. Figure 1 describes the experimental design and the order of the BTs.

### Chronic mild stress protocol

CMS is a series of unpredicted chronic mild stressors that intends to mimic humans’ everyday life stressors.^17^ The protocol is a 6-week-long exposure to unpredictable stressors (four stressors per day) including confinement to a restricted space (2 h), cage placement in a tilted cage (4 h), damp bedding housing (12 h), overcrowding six males in a standard housing cage (2 h), strobe light exposure (4 h), water deprivation for 12 h followed by exposure to an empty bottle for 1 h and replacing bedding for cold tap water (2 h). All stressors were imposed during the animal’s resting period (light phase of the diurnal cycle), except the damp bedding housing, which was done during the 12 h of the animal’s active period (dark phase of the diurnal cycle).

### Glucose tolerance test

Rats underwent i.p. glucose tolerance test (ipGTT) for insulin sensitivity assessment at three different time points throughout the experiment: before the start of CMS (week 0), in the middle of CMS (week 3) and at the end of the CMS protocol (week 6). Rats were on food deprivation for 16 h before the test, starting at the beginning of the dark phase of the light cycle, at 2000 hours. An i.p. glucose (Merck Millipore, Darmstadt, Germany) injection (2 g kg^−1^ body weight) forced a rapid glucose challenge and the glucose level in the blood was measured at time 0 (before the injection (G0)) and at 20, 40, 60 and 120 min post injection.^18^ Each time a drop of blood was collected through a small puncture close to the tip of the rats’ tail, and the glucose level was determined by a glucose meter device (One touch UltraMini, LifeScan, Madrid, Spain).

### Forced swimming test

Depression-like behavior was determined by assessing the animal’s learned helplessness behavior in the FST. Briefly, 24 h after a pre-test session (8 min), rats were individually placed in transparent containers filled with water (25 °C) to a depth such that they had no solid support for 5 min. Recorded videos were later scored by an investigator blind to the experimental groups. Learned helplessness behavior was defined as the amount of immobility time (defined as the time spent either immobile or making righting movements to stay afloat).^19^

### Elevated plus maze

Anxiety-like behavior was investigated by the EPM test. The apparatus is an elevated (72.4 cm above the floor) plus shaped platform (ENV-560; MedAssociates, St Albans, VT, USA) that contains two opposite open arms (50.8 × 10.2 cm) and two closed arms (50.8 × 10.2 × 40.6 cm). Rats were placed individually in the center of the apparatus and allowed to explore the maze for 5 min while its ambulation was monitored online with an infra-red photobeam system (MedPCIV, MedAssociates). The ratio of time spent in the open arms versus in the close arms was considered an index of anxiety-like behavior.

### Morris water maze

Cognitive function was assessed by the animals’ performance in spatial working, reference memory and reverse learning tasks in the MWM.^20^ The MWM apparatus was a circular container (170 cm diameter; 50-cm deep) filled with water (23 °C, around depth of 30 cm) in a dimly lit room. The container was divided into imaginary quadrants with an extrinsic visual clue for each, placed outside of the tank, and a small hidden platform (submerged 2 cm below the surface of the water) placed in the center of one of them. Data were collected using a video-tracking system (Viewpoint, Champagne au Mont d’Or, France).

The spatial working memory task was performed in 4 sequential days (four trials per day, maximum of 2 min per trial). Test sessions started, in each trial, by placing the rat in one of the imaginary quadrants (facing the wall of the tank) and ended when the animal reached the platform or when 2 min had passed (thereafter the animal was gently guided to the platform). Each trial corresponded to a different quadrant. The position of the platform was changed on each day, such that after 4 days it had been placed on all quadrants. The distance traveled to reach the platform (escape latency) was evaluated.^20, 21^ Immediately after the spatial working task, animals were tested for spatial reference memory for additional 3 days with the platform remaining in the same quadrant as that of the last day of the spatial memory task. This was to ensure that the animals correctly learned the position of the platform before the reversal learning task (further confirmed in the probe test). The test was performed as described above for the working memory task. After the last day of reference memory evaluation, the platform was positioned in the quadrant opposite to the previous one and rats performed the four-trial paradigm as described above. Thereafter the platform was removed and the animal was placed in the tank and allowed to search for the platform for 2 min. The distance traveled in the quadrant containing the final positioned platform ('target') versus the total distance traveled was calculated. In the water maze paradigm, to evaluate working memory, the daily trial-to-trial progression of the distance swum to reach the platform was averaged for the different platform locations and, in the reference memory, day-to-day progression was averaged across the four daily trials for the same platform location.

### Corticosterone determinations

To assess the glucocorticoid profile, blood samples were collected from each subject in two time points, namely light phase (0900–1000 hours) and dark phase (2100–2200 hours). A few drops of blood were collected by lancing the tip of the tail. The collected sera was stored at −80 °C until analysis. Blood collections were performed 5 days after the last BT was completed to diminish any influence of the tests on the glucocorticoid levels. Serum corticosterone levels were determined by radioimmunoassay (MP Biochemicals, Costa Mesa, CA, USA).

### Statistical analyses

Sample size determination was based on the main statistical procedures used in this manuscript. For the different procedures a medium or high effect size, a type I error *α*=0.05 and a statistical power (type II error) of 0.8, were considered. A total of 42 animals were used in each independent set of experiments. Statistical test assumptions were validated for all the analysis performed. Normality was assessed by Kolmogorov–Smirnov test and confirmed by the evaluation of skewness and kurtosis. The analysis indicated that when normality was not accomplished all variables presented absolute skewness and kurtosis below 1. This suggests that the distribution was close to normal, thus parametric tests for statistical analysis were used. Sphericity assumption (for repeated measures) and homogeneity of group variances were also verified and statistical analyses were made accordingly. Multiple linear regression models (enter method—all variables entering the regression model at the same time) were used to identify the main predictors of body weight gain. Mixed-design analysis of variance was used considering between (two factors: diet and CMS) and within subject factors for weekly measurements of food intake, distance swum in each trial in the spatial working task, distance swum in each day in the reference memory performances in the MWM and glucose level in each time point in the ipGTT. The data from the EPM, FST, probe test of MWM, corticosterone levels and the different time points in the diurnal circadian rhythm of food intake were analyzed by two-way analysis of variance considering CMS and diet as between subject factors. When there was an interaction effect between groups, it was decomposed by splitting the samples according to either diet or CMS, and independent samples *t*-test was performed. Outliers were excluded based on the *Z*-score criteria *Z*>│3│. Results are expressed as group mean±s.e.m. and statistical significance was accepted for *P*<0.05 (two-sided). Statistical analysis was performed using IBM SPSS statistics 20.0 (IBM, Armonk, NY, USA).

## Results

### CMS and HFD modulate body weight curves and feeding behavior

**Figure 2:**
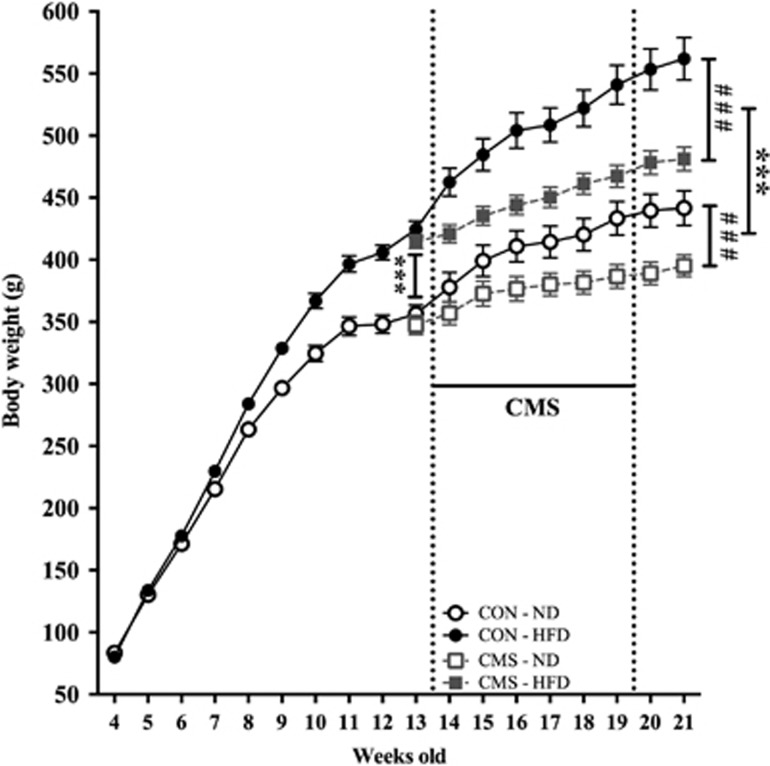
Body weight gain throughout the experiment. (*n*=11 each control group, *n*=8 CMS-ND group, *n*=12 CMS-HFD group; *** and ^###^: *P*<0.001. Data presented as mean±s.e.m.). *** Shows diet effect and ^###^ indicates CMS effect. CMS, chronic mild stress; HFD, high-fat diet; ND, normal diet.

Weekly analyses of the body weight gain and calorie intake were analyzed separately in two distinct periods: (i) exposure to different diets to the beginning of CMS (4–13 weeks of age); and (ii) during CMS (14–21 weeks of age). In the first step of the analysis, multiple linear regression models were used to explore the diet effect on body weight at the week 13, controlling for initial weight (week 4). Thereafter, body weight at week 21 of age was considered as the dependent variable, while diet, stress and body weight in the week 13 were considered as predictors. Multiple linear regression analysis of the first period was statistically significant (F_(2,39)_=26.91; *P*<0.001), indicating that 56% (*R*^2^=0.58; *R*^2^_Adj_=0.56) of the body weight at week 13 was explained by diet (*β*=0.77; *P*<0.001) and by initial body weight (*β*=0.19; *P*=0.08). The regression analysis was also significant at 21 weeks (F_(3,38)_=61.66; *P*<0.001), showing that 82% (*R*^2^=0.83; *R*^2^_Adj_=0.82) of the body weight at week 21 was significantly explained by stress (*β*=−0.30; *P*<0.001), body weight (*β*=0.65; *P*<0.001) and diet (*β*=0.23; *P*=0.038) on week 13 (Figure 2). This indicates that after controlling for diet and body weight on week 13, stress has a significant effect on body weight at week 21.

**Figure 3:**
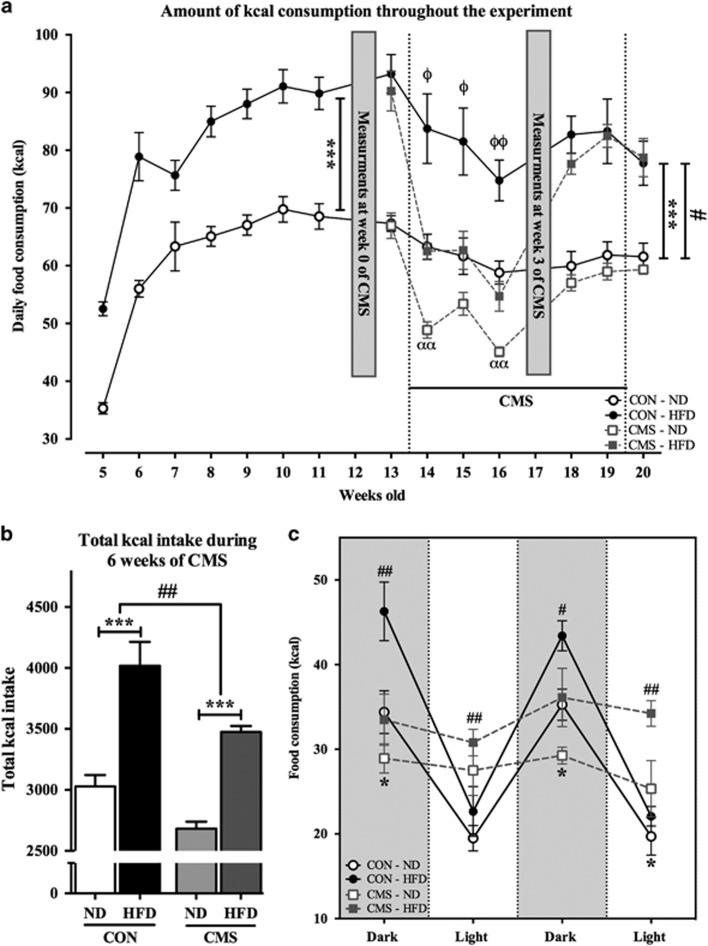
Food consumption measurements (^ϕ,^ *: *P*<0.05; ^ϕϕ, ##,^ and αα: *P*<0.01; ***: *P*<0.0001. * and ^#^ indicate the overall effect of diet and CMS, respectively). Data presented as mean±s.e.m. All the measurements are shown as kcal consumption per animal. (**a**) Energy intake by kcal per day throughout the experiment. (*n*=5 cages for each CON group, *n*=4 cages CMS-ND group, *n*=6 cages CMS-HFD group). * and *** present CMS and diet overall effect, respectively. Statistically significant differences between CON and CMS groups are shown by ^ϕ, ϕϕ^ for ND and αα for HFD. (**b**) Total kcal intake during the 6 weeks of CMS. (**c**) Food consumption evaluation in separate phases of the diurnal circadian rhythm, measurements were assessed at the time of phase change (0800 and 2000 hours) (*n*=4 cages for each group); * and ^##^ indicate the overall effect of diet and CMS, respectively. CMS, chronic mild stress; CON, control; HFD, high-fat diet; ND, normal diet.

Statistical analysis for daily food consumption (in kcal) was also separated in the same two analysis steps previously described (Figure 3a). The first analysis indicated that groups fed with HFD consumed higher amounts of kcal compared with the ND group (F_(1,12)_=36.2; *P*<0.001), a pattern that remained unaltered in the second phase (F_(1,8)_=26.6; *P*=0.001) regardless of exposure to CMS. However, in both CMS groups a remarkable drop in the amount of energy intake in the first 3 weeks of the CMS protocol when compared with their CON group (CMS effect on, week 14: F_(1,14)_=21.6; *P*<0.001; week 15: F_(1,15)_=9.98; *P*=0.006; week 16: F_(1,15)_=35.84; *P*<0.001) was noted. This reduction disappeared after the third week, as the energy intake reached the control level by the fourth week of CMS. Nevertheless, there was a CMS (F_(1,16)_=9.49; *P*=0.007) and diet (F_(1,16)_=56.4; *P*<0.0001) effect in the total amount of energy consumed during the 6 weeks of CMS. Further analysis when the CMS effect was considered independently of the diet, indicated that energy intake of the CMS-HFD group was significantly lower than that of the CON-HFD group (*t*_(9)_=2.9; *P*=0.02), a finding that was not seen in the CMS-ND versus CON-ND groups (*t*_(7)_=1.53; *P*=0.17) (Figure 3b).

In addition, data from food intake in separate light phases indicated a circadian rhythm in the CON groups with a higher amount of consumption during the activity period (dark phase) compared with the resting period (light phase). This circadian pattern was remarkably flattened in both stressed groups, which led to an equally distributed amount of energy intake throughout the diurnal cycle (Figure 3c) (diet effect: in dark phases, (F_(1,10)_=9.1; *P*=0.01) and (F_(1,10)_=9.9; *P*=0.01), respectively; in light phases, (F_(1,11)_=1.66; *P*=0.22) and (F_(1,11)_=6.3; *P*=0.03), respectively. CMS effect: in dark phases, (F_(1,10)_=11.2; *P*=0.01) and (F_(1,10)_=7.8; *P*=0.02), respectively; in light phases, (F_(1,11)_=10.47; *P*=0.01) and (F_(1,11)_=15.68; *P*=0.002), respectively).

### HFD and CMS induce glucose intolerance

**Figure 4:**
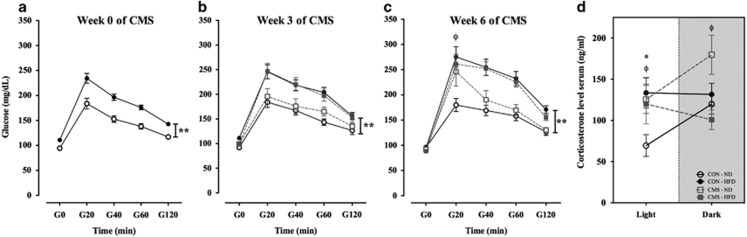
Glucose tolerance test (GTT) and corticosterone levels. (**a**) GTT in week 0 (before the start of CMS), (**b**) GTT in week 3 (half way through CMS), (**c**) GTT in week 6 (at the end of CMS). ** presents the statistically significant diet overall effect between groups. ^ϕ^ indicates the statistically significant increase of glucose level in the CMS-ND when compared with CON-ND. (*n*=11 each control group, *n*=8 CMS-ND group, *n*=12 CMS-HFD group; * and ^ϕ^: *P*<0.05 and **: *P*<0.01. Data presented as mean±s.e.m.) (**d**) Corticosterone levels in the light (0900 hours) and dark (2100 hours) phases of diurnal light cycle after 6 weeks of CMS protocol. Data illustrate an interaction effect in both light- and dark-phases corticosterone levels. ^ϕ^ indicates the statistically higher level of corticosterone in CMS-ND group when compared with CON-ND group. * indicates the statistically higher level of corticosterone in CON-HFD group when compared with CON-ND group. (*n*=6 for each group; * and ^ϕ^: *P*<0.05. Data presented as mean±s.e.m.). CMS, chronic mild stress; CON, control; HFD, high-fat diet; ND, normal diet.

ipGTT was performed in three time points during the CMS protocol (Figure 4). Glucose levels 20 min after glucose injection (G20) displayed an increase in all groups, as expected. However, there was a glucose tolerance impairment in the HFD groups, with a significant diet effect between groups in all time points: week 0 of CMS (F_(1,40)_=33.07; *P*<0.001) (Figure 4a); week 3 of CMS (F_(1,36)_=22.47; *P*<0.001) (Figure 4b); and, week 6 of CMS (F_(1,37)_=25.23; *P*<0.001) (Figure 4c). Furthermore, within ND group, CMS animals presented a higher peak compared with the CON group at G20 at the end of week 6 of CMS (*t*_(14)_=−2.22; *P*=0.043). This impairment was not observed in the HFD group, probably due to the ceiling effect of the diet in this time point. Interestingly, the impairment noted in the CMS-ND group was not yet present at week 3 of CMS.

### Corticosterone serum levels are disrupted by CMS and HFD

Glucocorticoid data showed an interaction effect in the corticosterone levels (F_(1,23)_=4.7; *P*=0.04) when the blood sample was collected in the light phase; however, no CMS or diet effect was noted between groups. These data indicates that CMS-ND had significantly higher glucocorticoid levels compared with their CON group (*t*_(10,7)_=−2.6; *P*=0.025). On the other hand, in the HFD groups no statistical significant difference was observed between HFD-CON and HFD-CMS groups (*t*_(11,12)_=−0.9; *P*=0.4). Data from the dark phase displayed an interaction (F_(1,22)_=8.4; *P*=0.008) and a diet effect (F_(1,22)_=4.6; *P*=0.043) in between groups. Additional analysis revealed a similar result, namely significantly different glucocorticoid levels in the ND group (*t*_(10)_=−2.5; *P*=0.048) but not in the HFD group (*t*_(12)_=1.7; *P*=0.11). CON-HFD animals display increased corticosterone levels when compared with the CON-ND animals (*t*_(11)_=−2.7; *P*=0.02) (Figure 4d). Also of notice, CON-ND display the expected increase in the corticosterone levels in the dark phase when compared with those in the light phase (*t*_(5)_=−4.54; *P*=0.006), an effect that was blunted in the CON-HFD group (*t*_(6)_=0.1; *P*=0.93).

### CMS and HFD alter emotional and cognitive behaviors

**Figure 5:**
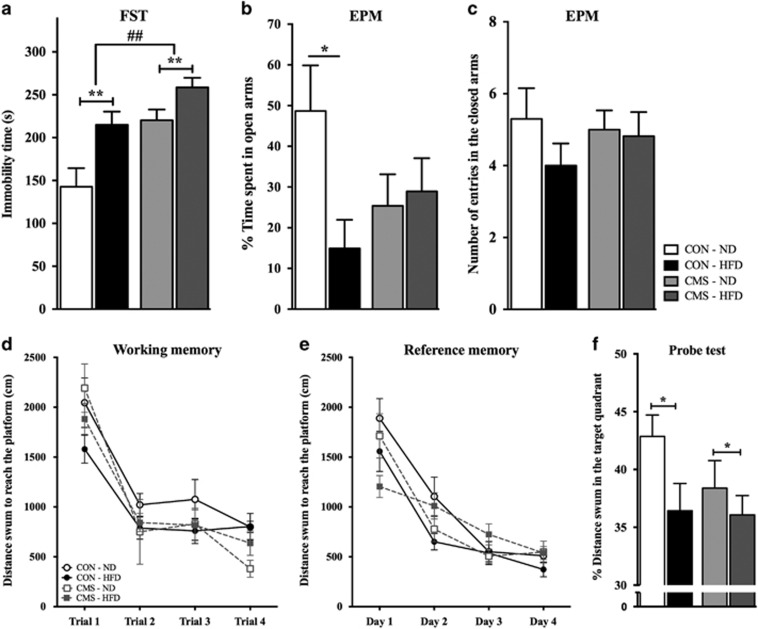
Behavioral assessment. (**a**) Forced swimming test (FST). Learned helplessness assessment by the time of immobility in the 5 min of testing. (**b**) Elevated plus maze (EPM). Anxiety-like behavior evaluation by the time spent in the open arms. There is an interaction effect between diet and CMS between groups. (**c**) EPM. Number on entries in the open arms indicate the locomotor activity. (**d**) Spatial working task. Each trial shows the average of the distance swum to reach the platform in the same trial in all 4 days of the test, which illustrates the learning progression. (**e**) Reference memory. Each day indicates the average distance swum in the 4 trials of the same day to evaluate the speed in learning the position of the platform. (**f**) Probe test. Distance swum in the quadrant of the new positioned platform after the reference memory task indicates the animal’s behavioral flexibility. For all the figures (*n*=11 each control group, *n*=8 CMS-ND group, *n*=12 CMS-HFD group; *: *P*<0.05; ** and ^##^: *P*<0.01. Data presented as mean±s.e.m.). * and ** indicate diet and, ^##^ indicates CMS overall effects between groups. CMS, chronic mild stress; HFD, high-fat diet; ND, normal diet.

FST data showed that both diet (F_(1,27)_=11.1; *P*=0.003) and CMS (F_(1,27)_=12.56; *P*=0.001) had an overall effect on immobility time (Figure 5a), indicating a significantly increased immobility time in animals exposed to a HFD, and CMS when compared with CON-ND.

EPM data indicated no remarkable effect of diet or of CMS on anxiety-like behavior. On the other hand, an interaction effect between diet and CMS was observed in all groups, showing that animals with HFD and CMS spent less time in the open arms when compared with the CON-ND (F_(1,36)_=4.6; *P*=0.039) (Figure 5b). Further analysis also indicated that CON-HFD spent significantly less time in the open arms compared with CON-ND (*t*_(18)_=2.65; *P*=0.02). In addition, the number of total entries in the closed arms, which is an index of locomotor activity, was similar in all groups (diet effect: (F_(1,34)_=1.1; *P*=0.31); CMS effect: (F_(1,34)_=0.1; *P*=0.72)) (Figure 5c).

The learning curve in the MWM task was not different between groups, both for the spatial working (diet effect: (F_(1,34)_=3.08; *P*=0.09); CMS effect: (F_(1,34)_=1.38; *P*=0.25)) (Figure 5d) and reference memories (diet effect: (F_(1,36)_=3.59; *P*=0.07); CMS effect: (F_(1,36)_=0.08; *P*=0.78)) (Figure 5e). However, the probe test showed that diet had a significant overall effect on the distance swum in the target quadrant (F_(1,35)_=4.31; *P*=0.045) (CMS effect: (F_(1,35)_=1.28; *P*=0.26)), with animals on HFD swimming a smaller distance in the 'target' quadrant when compared with their corresponding ND group (Figure 5f).

## Discussion

Summarily, in this study we showed that HFD consumption, besides leading to obesity as expected,^14^ altered the animals’ behavioral state by promoting depressive- and anxiety-like behaviors. The diet-induced body weight increase was particularly evident by the end of the experiment (5 months of age), with the CON-HFD animals weighting 30% more than the CON-ND. A delayed body weight gain of animals on ND is a well-known effect of the CMS protocol.^13, 22, 23, 24^ Interestingly, exposure to CMS did not further aggravate the HFD-induced behavioral changes, while stress reduced the amount of daily food consumption; regardless of the type of diet this reduction was only observed in the first 3 weeks of exposure to CMS. The initial immediate decrease of food intake observed on CMS exposure is identical to that reported on previous studies on acute restraint stress,^25^ but here it is also now reported that 3 weeks after the CMS protocol, animals recover their normal pattern on energy consumption.

Of note, both in CMS-ND and in CMS-HFD the body weight gain was reduced when compared with that of the CON group. Furthermore, despite of similar trends, the total amount of energy intake in the 6 weeks of the CMS period was significantly smaller in the CMS group fed by HFD when compared with the CON-HFD; an alteration that was not observed in the ND group (CMS-ND versus CON-ND). These interesting findings suggest that the CMS-HFD group lost weight due to a reduction in energy intake during the extension of the protocol, while in the CMS-ND group other factors probably underlie the reduction in the body weight gain. These factors may include different metabolic and hormonal milieu of normal, obese and stress-exposed individuals. In fact, several publications have reported the deleterious impact of obesity and of stress in feeding habits, blood and brain inflammation, and in neurodegeneration.^1, 26^ For instance, white-adipose tissue itself produces adipokines,^27^ interleukins and leptin (an adipokine regulating food intake through dopamine pathway).^28^ Hence, high levels of white-adipose tissue will secrete higher levels of adipokines directly modulating feeding habits and the inflammatory profile. This is potentiated in animals with obesity due to the hyperplasia and hypertrophy of their adipocytes.^29^ On the other hand, stressful events can also trigger an inflammatory profile, since higher levels of corticosteroids and catecholamines can potentiate the production of cytokines in a similar manner to an inflammatory state, exerting a direct impact on behavior.^30^

Another interesting observation relates to the circadian rhythm of food consumption: rats normally consume remarkably more (peak) during their active period (dark phase) compared with the resting period. Here, this pattern was inverted in both CMS groups (increased food intake in the light-period and decreased in the dark phases of the daily light cycle). We have previously found that exposure to CMS in the light phase leads to forced awakening and circadian rhythm disruption.^31^ In accordance, forced activity in the light phase appears to modify the temporal pattern of food intake, potentially leading to a misalignment of metabolic function with the biological clock, the so-called internal desynchronization.^32, 33, 34^ Along with other complications, internal desynchronization causes behavioral instability, sleep disturbance and dampening of metabolic and endocrine diurnal rhythms. These, in the long-term, can lead to many disorders, such as metabolic syndrome and depression.^35, 36^

As a measure of metabolic alterations, ipGTT was used. Injection of glucose to fasted animals should cause a sudden rise of the serum glucose levels in G20, which then declines to the baseline values in ~2 h.^37^ Here, we observed a significant impaired glucose tolerance in the HFD groups in all three periods assessed. These data are in accordance with previous reports, indicating metabolic alterations in HFD-fed subjects, such as type 2 diabetes and insulin resistance.^14, 37, 38^ Interestingly, glucose tolerance impairment in the CMS-ND group occurred only at the end of the stress protocol, and not at week 3. This reveals that the metabolic alterations were only developed after the daily food intake recovery, suggesting the impact of the body’s distinct coping strategies on metabolism during the exposure to chronic stress, such as energy expenditure acceleration, that also leads to body weight reduction. In the HFD group, on the other hand, no further increase in glucose concentration was observed in the CMS group, which may be due to the ceiling effect induced by the HFD.

Furthermore, glucocorticoids, which are activated through the hypothalamic–pituitary–adrenal axis, are known modulators of energy homeostasis,^2, 39^ and hypothalamic–pituitary–adrenal axis over-activation has been shown to alter insulin action resulting in 'insulin resistance'.^39^ Accordingly, the present data showed that the CMS-ND group had significantly higher levels of glucocorticoid in both the light and dark phases of the diurnal cycle. Of notice, the HFD group displayed higher basal glucocorticoid levels, as previously reported,^2, 40^ which were not further elevated by CMS. In addition, when compared with the CON-ND, the CON-HFD group had increased corticosteroid levels in the light phase of the diurnal cycle, the time of the day in which the behavior tests were performed; thus diet alone is associated to an hormonal pattern that may underlie the observed behavior differences as will next be discussed.

Stress, especially when driven by CMS protocols, has a known impact on emotional behavior.^41^ Mood was assessed by the FST, which measures behavioral despair (learned helplessness).^7, 19, 41^ Importantly, this test was performed and not the sucrose-preference test (that measures anhedonia) as the animals were under distinct diets with different tastes and caloric value, which may have an impact on the animals food preferences. Here, regardless of body weight, a CMS overall effect in the immobility time was observed, as it has been demonstrated before.^41, 42^ Of notice, CON-HFD had an immobility time equal to that of the CMS-ND group, indicating a depressive-like behavior. Again of interest, exposure to CMS did not further increase the immobility time already observed in the CON-HFD group. In accordance, a direct effect of obesity on mood disorders, namely in depression, has been described in the literature,^43, 44, 45^ although there are some conflicting observations.^3, 4, 46^ A recent report on the effect of antidepressants in mice under a HFD and exposed to a stress protocol showed that mice fed with HFD (for 14 weeks) and exposed to CMS protocol (for 4 weeks) had a decreased body weight and increased immobility time in the FST. This result supports a potentiation of the depressive-like behavior in the CMS-HFD when compared with the CON-HFD group.^15^ Of notice, HFD rats in the present study showed a resistance to further increase the depressive-like phenotype in response to CMS. This can be due to the fact that animals were fed with HFD for a 4-week longer period before application of the CMS protocol, which may have more pronounced impact on the animals’ behavioral alteration in response to the stressors. Moreover, here rats rather than mice were used, and it is known that these species may display different behavioral profiles. Still, both studies revealed a depressive-like behavior outcome of animals under HFD. While the increased corticosteroid levels induced by HFD may contribute to the behavioral changes observed, additional research is necessary to understand the precise mechanisms of the effect of diet-induced obesity on the response to environmental stressors, especially given the known role of orexigenic and anorexigenic peptides in mood behavior.^25, 47^

Stress is also known to trigger anxiety,^16, 41, 42^ which was also observed here given the decrease in the time spent by stressed animals in the open arm of the EPM, regardless of the animals’ body weight. Animals fed with HFD also presented anxiety-like behavior when compared with those on ND, which is in accordance with observations reported in another study of diet-induced obese mice.^38^ Although there are inter-species differences between these studies, it is interesting that similar evidences are found in some behavioral dimensions. Moreover, the number of entries in the closed arms indicated that the CON-HFD group did not have compromised locomotor or exploratory abilities in this test. Interestingly, again, CMS did not further aggravate the anxiety-like behavior displayed by the CON-HFD group. These findings are in accordance with the glucocorticoid data, whose levels do not further increase in the HFD by the CMS.

With respect to the effect of CMS and HFD on cognition, no spatial learning or reference memory impairments were detected, which is in line with some previous observations.^31, 41^ However, some studies have found correlations between memory and learning impairments with other types of stress^7, 16, 20, 48, 49^ and with obesity.^50, 51^ Still, the present data reveals a remarkable diet effect in the distance swum in the target quadrant in the probe test, indicating disturbed behavioral flexibility in the animals fed by HFD.

In summary, the present study shows the interplay between HFD and chronic stress in different behavioral end-points, highlighting that animals with different metabolic and mood states respond differently to chronic exposure to mild stressors. This is a significant finding largely neglected in the field. Even though this is a descriptive study, and thus the underlying mechanisms need to be further explored, the data presented herein are of relevance particularly in the context of the current obesity epidemic, and indicates that HFD impacts on the response to CMS, which should be considered when addressing the consequences of obesity on behavior.
